# Factor V Leiden and Prothrombin 20210A Mutations among Turkish Pediatric Leukemia Patients

**DOI:** 10.1155/2012/250432

**Published:** 2012-02-16

**Authors:** Dilara Fatma Akın, Kadir Sipahi, Tuğba Kayaalp, Yonca Eğin, Serpil Taşdelen, Emin Kürekçi, Üstün Ezer, Nejat Akar

**Affiliations:** ^1^LOSEV the Foundation for Children with Leukemia, Ankara, Turkey; ^2^Department of Pediatric Genetics, Ankara University, Ankara, Turkey; ^3^Department of Pediatric Haematology, Gülhane Military Medical Academy, Ankara, Turkey; ^4^TOBB-ETU Hospital, Ankara, Turkey

## Abstract

This study was undertaken to determine the prevalence of the Factor V 1691 G-A and PT 20210 G-A mutations in Turkish children with leukemia. We genotyped 135 pediatric leukemia patients with for these mutations. Eleven (8%) of the 135 patients were heterozygous for the FV 1691 G-A mutation. Seven (5,1%) of the patients carried the PT 20210 G-A heterozygous mutation. Of the 135 patients, only three had thrombotic event, none of which had these two mutations, which is common in Turkish population. Our findings revealed a controversial compared to the previous reports, which needs further investigation.

## 1. Introduction

The reported incidence of thrombosis ranges from 2.4% to 11.5% and an important complication in pediatric acute lymphoblastic leukaemia (ALL) patients. Its occurrence may complicate the treatment course with a negative prognostic impact [2, 3].

Thromboembolic events (TEs) are thought to result from the interaction of various factors, including effects of disease itself, central venous line, and chemotherapy, catheterization, infections, dehydration, chemotherapeutic agents such as steroids and L-asparaginase (L-Asp), and acquired or inherited prothrombotic defects (IPDs) may influence the occurrence of thromboembolism [4–8]. 

Various molecular defects of different hemostatic components have been established as risk factors for thromboembolic diseases mainly in adults and pediatric cases such as deficiencies of protein C, protein S, and antithrombin, resistance to activated protein C, mostly due to the factor V (FV) G1691A gene mutation and the prothrombin (PT) G20210A genotype [9].

Chemotherapy can influence the haemostatic system either through the direct effect of the chemotherapeutic agent or through complications such as infections. Corticosteroids activate platelet function, asparaginase reduces the synthesis of natural anticoagulants and in combination they increase the risk of TE in children with ALL. Some studies have shown increased thrombin activation at diagnosis in children with ALL [6, 9]. 

Factor V gene G-A transition at nucleotide 1691 in exon 10 is the leading cause of constitutional thrombophilia and results in with thrombus formation and creates a protein that is resistant to APC in the majority of affected individuals. The risk of developing thrombotic episodes for persons heterozygous for the FVL mutation is about 5–10 fold and for those homozygous is 80–100-fold higher than the general population.The frequency of FVL is about 8% in our healthy population [10]. 

The prothrombin 20210 G-A polymorphism is the second most common inherited risk factor for thrombophilia. The polymorphism is located at position 20210 in the 3′ untranslated region of the prothrombin gene and is caused by single base change G-A. Carriers of the rare A allele have higher prothrombin levels than carriers of the G allele and a higher risk of venous thrombosis [11, 12]. The frequency of PT 20210 is 2.6% in our healthy population [13].

Several studies on genetic risk factors for thrombosis excluded those patients with cancer-related thrombosis. Although surgery, chemotherapy, central venous catheters, and systemic hypercoagulability were associated with venous thromboembolism (VTE) in cancer, previous reports on the association of FVL and/or prothrombin 20210A with cancer-associated VTE are few and present conflicting results [14–18]. 

Recent studies reported that the Factor V Leiden mutation does not play a significant role on the overall incidence of thrombosis that occurs in children with cancer. The North American Prophylactic Antithrombin Replacement in Kids with Acute Lymphoblastic Leukemia Treated with Asparaginase (PARKAA) study failed to show any correlation between the presence of the PT 20210A variant or the FV G1691A mutation and development of asymptomatic VTE [15]. A study by Mitchell et al. reported that none of 22 children with TEs were positive for factor V Leiden or prothrombin gene 20201A but 22 of 60 children had TEs, a prevalence of 36.7% without this mutation [19]. 

Nowak-Göttl et al. reported in a prospective multicenter study which focused on the role of prothrombotic risk factors in consecutively admitted newly diagnosed children with ALL carrying prothrombotic risk factors. In this study 32 of 289 consecutively admitted leukemic children (11%) treated according to the ALL-BFM 90/95 study suffered venous thromboembolism [20]. Caruso et al. demonstrated that inherited thrombophilia increased the risk of VTE in pediatric ALL patients by approximately 8.5 (4.4–17.4). They indicated that a meta-analysis of prospective studies in childhood ALL and VTE found that symptomatic VTE was diagnosed in the central nervous system (CNS) in 50% of cases, venous VTE in 31%, and cerebral infarction or stroke in 18% [3]. 

As several previous reports had conflicting data on common thrombophilic mutations in leukemic children, we screened our patients with pediatric leukemia for these two mutations.

## 2. Patients and Methods

This study population consisted of 135 patients 78 males and 57 females, aged between 1 and 15 years who were admitted to our hospital with the diagnosis of pediatric leukemia.114 had ALL, 13 had AML, 6 had chronic myelocyctic leukemia (CML), and 1 patient had mixed leukemia, 1 patient acute promyelocytic leukemia (APL).

An informed written consent was obtained from all the patients' parents. Blood samples were collected with EDTA-containing tubes and DNA was extracted from peripheral blood leukocytes according to phenol-chloroform method. FV 1691 G-A and PT 20210 G-A mutation was analysed by real-time PCR (RT-PCR).

Genotyping of FV 1691 G-A, PT 20210 G-AT polymorphisms was screened with real-time PCR using fluorescence melting curve detection analysis by means of the Light Cycler System (Roche Diagnostics, Manheim, Germany). For FV 1691 G-A, forward primer: 5′-TGCCCAGTGCTTAACAAGACCA-3′; reverse primer: 5′-CTTGAAGGAAATGCCCCATTA-3′; anchor hybridization probe: 5′-LC-Red705 TGTCCTTGAAGTAACCTTTCAGAAATTCTG-3′-PHO; mutation probe: 5′-GGCGAGGAATACAGGTAT-3′. For prothrombin, forward primer: 5′-CCGCTGGTATCAAATGGGG-3′; reverse primer: 5′-CCAGTAGTATTACTGGCTCTTCCTG-3′, anchor hybridization probe: 5′-LC-Red640-TCCCAGTGCTATTCATGGGC-3′-PHO; mutation probe: 5′-CTCAGCGAGCCTCAATG-3′.

Melting point analysis was performed according to the instructions of the company.

## 3. Statistical Analysis

The chi-square test was used to compare categorical variables. *P* value of <.05 was considered statistically significant. Allelic frequencies were calculated by gene-counting method and the genotype distribution with Hardy-Weinberg expectations by a *x* ^2^ and Fisher's exact tests.

## 4. Results

Genotype distributions of FV 1691 G-A, PT 20210 G-A were given in Table 1, respectively.

 Eleven of the 135 patients were heterozygous for the FV 1691 G-A mutation. Seven of the patients carried the PT 20210 G-A heterozygous mutation. FV 1691A homozygous mutant genotype was found in 3 of the 135 patients and homozygous PT 20210A mutant genotype was not detected in 135 patients. None of the patients carried both mutations. In childhood leukemia patients group allelic distributions for FV 1691 G is 0,93%, A allele is 0,06% and PT 20210 G is 0,97% and A allele is 0,02%. Heterozygosities of FV 1691 G-A substitution demonstrate a difference between childhood leukemia patients and healthy newborns [(OR: 1.25/0.6–2,4 CI (95%); *P*: 0,03)] (Table 2).

Three (2,2%) of the 135 patients had thromboembolism. Sinus thrombosis, the cerebral ischemia, and deep vein thrombosis were the diagnosis of these cases. Neither of these three patients had thrombophilic mutations. 

## 5. Discussion

This study was undertaken to determine the prevalence of the Factor V 1691 G-A and PT 20210 G-A mutations in Turkish children with leukemia. These two mutations have been shown to be the most common heritable risk factor predisposing to venous thrombosis in adults [21–23] and also in the Turkish population [10, 24]. 

In this study, we have shown that Factor 1691 G-A and PT 20210 G-A mutations are not associated with the development of thrombosis in our childhood leukemia patients. Other studies reported controversial data on PT than FV mutations [9, 14, 15]. The prevalence and the pathogenesis of thrombosis associated with ALL are obscure. The primary disease itself can activate blood coagulation via procoagulant substances or by impairment of fibrinolytic or anticoagulant pathways. Additionally, chemotherapy and prothrombotic risk factors of the host may play a contributory role. Epidemiologic studies of pediatric thrombosis in leukemia have been greatly emphasized by small numbers, making estimates of thrombosis risk in this condition very difficult [3, 14, 25, 26]. The preponderance of the data, including ours, suggests no association of FVL and PT 20210A with leukemia-related thrombosis. Anticoagulant therapy initiated to all patients, which may be the reason for low prevelence of thrombosis. 

In conclusion, our study suggests that the FV 1691A and the Prothrombin 20210A mutations are not associated with TE in pediatric leukemias. 

## Figures and Tables

**Table 1:** Genotype distributions in patients for FV 1691 G-A and PT 20210 G-A.

Polymorphism FV 1691 G-A	Genotype distributions *n*: 135 (%)	Polymorphism PT 20210 G-A	Genotype distributions *n*: 135 (%)
G/G	121 (89,6%)	G/G	128 (94,8%)
G/A	11 (8%)	G/A	7 (5,1%)
A/A	3 (2%)	A/A	—

**Table 2:** Comparison of FV 1691 G-A in between childhood leukemia and healthy newborns gruops.

Polymorphism FV 1691 G-A	Childhood leukemia *n*: 135 (%)	Newborns* *n*: 551 (%)	OR CI (95%)	*P*
G/G	121 (89,6%)	494 (89,6)	1	
G/A	11 (8%)	56 (10,1%)	1,25 (0,6–2,4)	0,5
A/A	3 (2%)	1 (0,1%)	0,08 (0,008–0.7)	0,03

Reference: *[1].
